# GW501516, a PPAR-BETA/DELTA agonist, improves inflammatory pathways in the kidney of high-fructose fed mice

**DOI:** 10.1186/1758-5996-7-S1-A121

**Published:** 2015-11-11

**Authors:** D'Angelo Carlo Magliano, Isabele Bringhenti Sarmento, Vanessa de Souza Mello, Carlos Alberto Mandarim de Lacerda, Marcia Barbosa Aguila

**Affiliations:** 1 Universidade do Estado do Rio de Janeiro, Rio de Janeiro, Brazil

## Background

Angiotensin-II type 1 receptor (AT1r) high activation is closely linked to a low-grade inflammation and oxidative stress that yield impaired renal function and, consequently, chronic kidney disease (CKD).

## Objectives

Therefore, the aim of this study was to verify if GW501516 could improve damage in the kidney of mice with high activation of AT1r.

## Materials and methods

To induce high activation of this receptor, mice were fed a high-fructose diet for eight weeks. The control group only received standard-chow (SC). After, the animals were randomly divided into four groups and the administration of GW501516 started and lasted three weeks. Morphological variables and urinary and plasmatic determinations were assessed. Renin and angiotensin converting enzyme (ACE)/AT1r axis protein and gene expression were evaluated as well as inflammatory cytokines and proteins. Also, the protein and gene expression of the antioxidant enzymes were verified.

## Results

GW501516 activated PPAR-beta/delta and its target genes PDK4 and CPT-1. Despite showing no effects either on ACE/AT1r axis or renin expression, GW501516 improved the inflammatory state in the kidney. It elicits an expressive reduction in the expression of inflammatory genes such as IL-1β, IL-6, MCP-1 and Cd68, irrespective of AT1 downregulation. However, no differences were found in oxidative stress.

## Conclusions

We conclude that GW501516, a PPAR-beta/delta agonist, acts downstream AT1r activation, improving inflammatory pathways in the kidney of high-fructose fed model.

